# The Impact of Visual Guided Order Picking on Ocular Comfort, Ocular Surface and Tear Function

**DOI:** 10.1371/journal.pone.0157564

**Published:** 2016-06-17

**Authors:** Angelika Klein-Theyer, Jutta Horwath-Winter, Dieter Franz Rabensteiner, Gerold Schwantzer, Georg Wultsch, Haleh Aminfar, Andrea Heidinger, Ingrid Boldin

**Affiliations:** 1 Department of Ophthalmology, Medical University of Graz, Graz, Austria; 2 Institute for Medical Informatics, Statistics and Documentation, Medical University of Graz, Graz, Austria; 3 AMEZ Graz occupational health centre, Medical University of Graz, Graz, Austria; Medical University Graz, AUSTRIA

## Abstract

**Purpose:**

We investigated the effects of a visual picking system on ocular comfort, the ocular surface and tear function compared to those of a voice guided picking solution.

**Design:**

Prospective, observational, cohort study.

**Method:**

*Setting*: Institutional. *Study Population*: A total of 25 young asymptomatic volunteers performed commissioning over 10 hours on two consecutive days. *Main Outcome Measures*: The operators were guided in the picking process by two different picking solutions, either visually or by voice while their subjective symptoms and ocular surface and tear function parameters were recorded.

**Results:**

The visual analogue scale (VAS) values, according to subjective dry eye symptoms, in the visual condition were significantly higher at the end of the commissioning than the baseline measurements. In the voice condition, the VAS values remained stable during the commissioning. The tear break-up time (BUT) values declined significantly in the visual condition (pre-task: 16.6 sec and post-task: 9.6 sec) in the right eyes, that were exposed to the displays, the left eyes in the visual condition showed only a minor decline, whereas the BUT values in the voice condition remained constant (right eyes) or even increased (left eyes) over the time. No significant differences in the tear meniscus height values before and after the commissioning were observed in either condition.

**Conclusion:**

In our study, the use of visually guided picking solutions was correlated with post-task subjective symptoms and tear film instability.

## Introduction

Computer-aided manual order picking solutions are widely used, primarily in pick-by-voice systems. However, the development of near eye display devices has created myriad new opportunities. One such opportunity is the use of manual sorting systems that visually guide operators in the picking process.

Although pick-by-voice systems are proven technologies and commonly used for commissioning of orders, visually guided picking system technology are currently still in an experimental stage. Nevertheless, the implementation of near-eye display devices is promising for the future of order picking systems and also in various other workplace scenarios. However, the workload associated with the use of a visually guided commissioning system has not yet been investigated. This system require workers to wear a headset with a see-trough display for one eye, enabling the transmission of information by augmenting visual perception via a projection in the field of vision. With either visual or voice guidance operators pick the required products from multiple warehouse locations with electric pallet stackers, driving at speeds of up to 45 km/h. The purpose of this study was to investigate ocular comfort, ocular surface and tear function parameters before and after the completion of a task using either a visual- or a voice-guided picking solution.

## Participants and Methods

A total of 25 volunteers aged between 25 and 39 years (median 29.5) were recruited into this study. The cohort included male subjects only. Written informed consent was obtained from each participant prior to data collection. The study was approved by the institutional review board of the Medical University of Graz, Austria.

All subjects were asymptomatic, did not wear contact lenses, had normal ranges of accommodation and did not have any type of ocular disorder. None of the subjects used eye drops or were taking any systemic medication. The temperature and humidity of the room were controlled within the ranges of 17–20°C and 60–70% relative humidity at an illumination of 200 lux. Two order picking sessions, each lasting over 10 hours, took place on two consecutive days. In one session the subjects used a visually-guided system and in the other they used a voice-guided system. All the subjects took part in both sessions. In the visual-guided condition, the operators wore a head-mounted see-through display and received instructions through the display to their right eyes.

**Fig 1:**
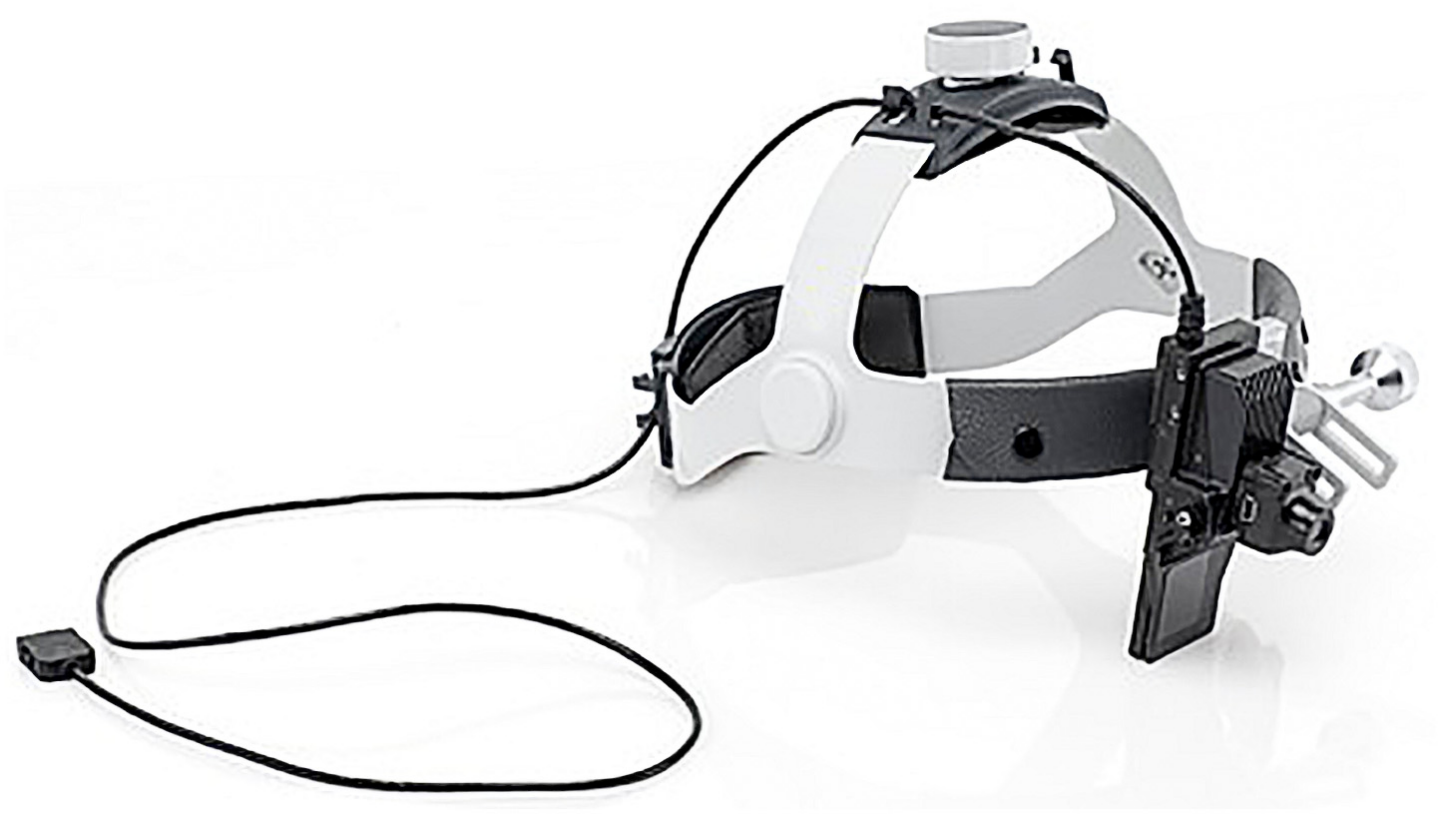
Visual guided system.

Knapp KiSoft Vision: See-through-display (SVGA 800*600 Pixel, up to 23mm distance to the eye), microcamera (800 x 480 Pixel, 30 frames per second). In this version the display was not changeable to the left side. “Fig 1”

**Fig 2:**
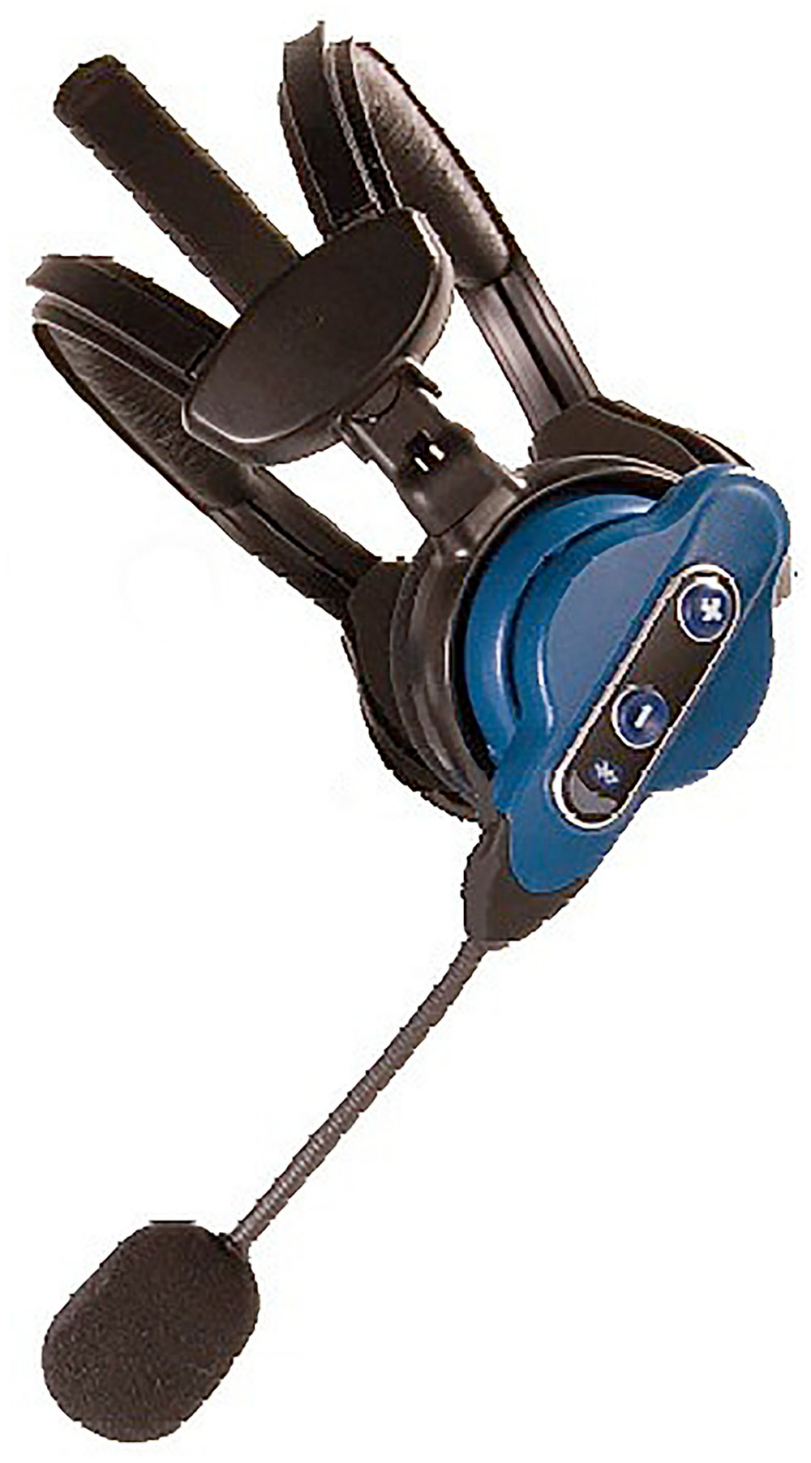
Voice guided system.

In the voice-guided condition, the operators used a wireless, wearable computer with a headset on both ears “Fig 2” and were instructed via the headset.

Subjective dry eye and asthenopic symptoms as well as objective ocular surface and tear function parameters of the participants were measured in a baseline examination and at the end of the commissioning sessions on each of the two days.

Subjective dry eye symptoms (i.e. the sensation of dry eye, ocular fatigue, ocular pain, foreign body sensation, irritation, burning sensation, and itchiness) of all participants were assessed using the tests described below.

The visual analogue scale (VAS) is a measurement instrument for subjective symptoms. We used a scale of 0–100. Each participant marked their level of ocular discomfort; 0 to indicate no symptoms and 100 to indicate severe discomfort. The VAS score is determined by measuring the distance between the left end of the line and the point marked by the subject.

The FACE scorecard scale ranges from 1–9. It shows images of faces with different expressions. For example, the saddest face (score 9) describes severe discomfort, and the happiest face (score 1) represents no symptoms.[1, 2]

The effect of working with these systems on asthenopia was tested with a questionnaire based on Stüdeli, 2001.[3] The original questionnaire was designed for visual display unit (VDU) users for investigation into eye discomfort, which is the second-most frequent problem reported by VDU operators.[4, 5]

Analyses of the objective parameters were performed non-invasively for both eyes with a Keratograph 5M (manufactured by Oculus GmbH, Wetzlar, Germany) in the order listed below.

The tear meniscus height was measured manually with an integrated ruler.

Tear film break-up time: the time from a complete blink to the initial tear film break-up was measured with the Keratograph. The pre- and post-task break-up times were recorded.

Redness of the conjunctiva: the bulbar redness (nasal, lateral, and total) and the limbal degree of redness (nasal, lateral, and total) were automatically and objectively measured and classified with the R-Scan module of the Keratograph 5M. The R-Scan module detects thin blood vessels in the conjunctiva and evaluates the degree of redness, using the following scoring scale: 0, no redness; 1, single conjunctival injections, 2, minor, scattered injections; 3, enhanced local injections; and 4, enhanced scattered injections.[6]

## Statistical Analysis

Tear meniscus height and conjunctival redness are described with mean and standard deviation (SD), BUT values were given as median with interquartile range (IQR) and subjective symptoms as median with range (minimum—maximum).

To quantify the changes in the subjective parameters during the working time, we calculated the differences between the baseline measurements and those taken after 10 hours of commissioning. These changes in the subjective parameters are given as mean with SD.

For the subjective symptoms, the differences between the baseline and 10 hours measurements and the differences in the amounts of change were assessed with the Wilcoxon signed-rank test.

For the objective parameters, we performed repeated-measures analyses of variances (rmANOVA) to test the effects of working time (baseline vs. 10 hours) and condition (vision vs. voice) as within-subjects factors and the possible interaction between the working time and the condition.

The rmANOVA of the BUT values was performed on rank-transformed data because all values over 20 seconds were taken as a single category.

P-values below 0.05 were considered statistically significant. All computations were performed with SPSS Statistics (Release 21.0.0.0 2012, International Business Machines Corporation, Armonk (NY), USA).

S1 File, supporting information regarding our main outcome measures

## Results

### Subjective Symptoms

In the vision condition, we found significantly higher VAS values at 10 hours of commissioning (median 5.0 [range 0.0–25.0]) than at baseline (median 3.0 [0.0–8.0]) (p = 0.026), but no significant differences were observed in the voice condition in the VAS values at baseline and at 10 hours of commissioning (2.0 [0.0–20.0] and 2.0 [0.0–18.0], respectively). These findings were associated with a significant difference in the increases in the VAS ratings, which were in mean 4.41 (SD 7.37) in the vision condition and were nearly nil (0.24 [SD 2.20]) in the voice condition (p = 0.045). Regarding the FACE scores and the Stüdeli values, we observed no differences between the conditions or the measurements (at baseline and at 10 hours of commissioning).

### Objective parameters

BUT values declined significantly (p < 0.001) in the visual condition (pre-task: 16.6 sec and post-task: 9.6 sec) in the right eyes that were exposed to the displays, the left eyes in the visual condition showed only a minor decline (pre-task: 18 sec and post-task: 16.5 sec) whereas the BUT values in the voice condition remained constant (right eyes) or even increased (left eyes) over the time.

We observed a statistically significant (p = 0.002) increase in the overall redness of the conjunctiva of the right eyes in both conditions after the working time and no significant difference between the conditions. The overall 10 hours measurement values of the right eyes were significantly higher than the baseline values in all subgroups (bulbar nasal right, p = 0.004; bulbar temporal right, p = 0.010; limbal nasal right, p = 0.001; and limbal temporal right, p = 0.019).

None of the redness of the conjunctiva values of the left eyes exhibited a significant effect in either condition.

No significant differences between conditions or pre/post-working time were observed in either eye in the tear fluid meniscus height values.

All results are summarized in Tables 1 and 2.

**Table 1:** Subjective Values.

	A	B
VAS		
baseline	3.0 (0.0–8.0)	2.0 (0.0–20.0)
10 hours	5.0 (0.0–25.0)	2.0 (0.0–18.0)
change	4.41 (7.37)	0.24 (2.20)
FACE		
baseline	1.0 (0.0–5.0)	2.0 (0.0–5.0)
10 hours	2.0 (0.0–5.0)	1.0 (0.0–4.0)
change	0.38 (.81)	-0.095 (1.34)
STÜDELI		
baseline	0.0 (0.0–0.1)	0.0 (0.0–0.2)
10 hours	0.0 (0.0–0.3)	0.0 (0.0–0.2)
change	0.0 (0.1)	-0.003 (0.078)

Values are median (minimum—maximum), except for change: values are mean (standard deviation)

A … Condition A-Vision

B … Condition B-Voice

**Table 2:** Objective values.

					ANOVA p-Values		
	A1	A2	B1	B2	A vs B	1 vs 2	IA
BUT sec							
ri	16.6 (6.7)	9.6 (7.3)	16.0 (6.0)	16.0 (5.0)	.004	.001	< .001
le	18.0 (6.0)	16.5 (4.5)	18.3 (5.0)	20.0 (5.0)	.037	.918	.214
Redness overall score							
ri	0.79 (0.25)	0.91 (0.26)	0.76 (0.21)	0.89 (0.25)	.563	.002	>.999
le	0.81 (0.35)	0.94 (0.35)	0.84 (0.28)	0.92 (0.28)	.875	.100	.629
Tear meniscus height mm							
ri	0.33 (0.10)	0.34 (0.10)	0.36 (0.17)	0.31 (0.08)	.959	.186	.303
le	0.32 (0.09)	0.34 (0.08)	0.36 (0.11)	0.33 (0.10)	.314	.962	.080

Values are mean (standard deviation) except for BUT (pseudometric): median (interquartile range IQR)

A … Condition A-Vision

B … Condition B-Voice

A vs B … p-Value for ANOVA main effect of condition (A-Vision vs B-Voice)

1 vs 2 … p-Value for ANOVA main effect of time (1,Baseline vs 2, 10 hours)

IA … p-Value for ANOVA interaction term (A1-B1)-(A2-B2)

ANOVA of BUT was carried out on rank transformed data

## Discussion

Ocular symptoms associated with the use of electronic devices, particularly at close working distances, are well known. Recent publications indicate that up to 90% of computer users experience ocular discomfort after prolonged computer use and approximately 10% of VDU users have severe complaints.[4,5,7,8] Although many factors influence this condition, the major contributor to ocular symptoms seems to be dry eyes.[9] Extensive investigations of eye problems and ocular symptoms of VDU workers have been undertaken. However, particular studies in regards to the tear film, the ocular surface, and comfort using near-eye display devices and augmented reality software have not yet been published. This was the focus of the present investigation.

Our study has three potential limitations due to the predefined study design. One is that the study population was small and consisted of only young, healthy males who were not contact lens wearers. Considering that females[10–14], subjects over the age of 30[10, 15], and contact lens wearers[10] seem to be at greater risk of developing dry eye disease when using VDUs, we have to assume that an average population might exhibit more symptoms and signs of ocular surface irritations than our study population. The second limitation is that we observed each condition over only a single day for a duration of ten hours. Although this time period exceeded a single typical working day, it would be interesting to investigate the prevalence of dry eye over longer periods of exposure (i.e. a few weeks). The third limitation is that we used a voice-guided system for the reference group. The task of order picking by voice with an electric pallet stacker that is driven at speeds of up to 45 km/h requires a high level of concentration and might itself cause ocular irritations. Due to these limiting conditions of the study, the expectation to find significant ocular signs and symptoms was rather low. Nevertheless, we found a significant increase in subjective symptoms and a significant reduction in the BUT values after the visually-guided commissioning work session.

The VAS values were significantly increased with the visual system when compared to the voice system and also with the visual system after the work session had finished (i.e. pre- vs post-task), which suggests that visually-guided picking solutions may adversely influence ocular comfort.

Monocular stimulation due to the monocular visual guiding system on one eye leads to binocular accommodation, asymmetric convergence and blurred far-distance vision[16]. In addition, retinal pictures on a single eye can also lead to fusion problems. These conditions might be a reason for a subjectively undefined ocular discomfort.

The analysis of the objective data revealed a significant decrease in the BUT values of the right eyes and a minor decrease in the values of the left eyes following the completion of the visually-guided picking task. The BUT values for the voice-guided condition remained stable (right eyes) or even increased (left eyes) after the work.

This decline in the BUT values after visually-guided picking might be related to a reduced blink frequency. The blink rate is known to be affected by the level of attention directed to a particular task.[17–19] It has been hypothesised that the excessive evaporation of tear fluid due to prolonged blinking intervals is a causative factor in VDU-associated dry eye. [20,21] Furthermore, not only the blink rate itself is presumed to decrease, phases of partial, incomplete blinks are also commonly observed during visual work at near distances.[20,22] However, one might expect a decrease in BUT values for both eyes, as the blink frequency is thought to be linked between the eyes. The left eyes showed only a minor decrease in BUT values after the visual guided picking task. It is therefore likely, that not only a decreased blink rate but also altered blink kinematics or other factors might have influenced the tear stability in the right eyes after the task of visually-guided picking in our study. Little is known about the complex impact of tasks of this nature on blinking and the ocular surface, which makes further studies on this topic warranted.

Uchino et al. [10] report in a study involving 672 Japanese office workers that dry eye disease in visual display terminal workers was observed to start with shortened BUT values that subsequently led to the development of subjective symptoms. Our results match this hypothesis with the findings of a significant decrease in BUT and a smaller but still significant increase in subjective symptoms post-task with the visually-guided system.

The results for the redness of the bulbar and limbal conjunctiva revealed increases in the overall redness of the right eyes after the task carried out with both guiding solutions. Irritation induced ocular hyperaemia mainly occurs in the interpalpebral fissure, where the ocular surface is exposed.[23] This finding may indicate that there are task related ocular irritations due to several factors and it might be reasonably assumed that both picking solutions may have an adverse influence, especially when performed on two consecutive days.

According to Gartner research (Gartner, Inc. Corporate Headquarters: 56 Top Gallant Road, Stamford, CT 06902 USA), there is a clear trend that will lead to the implementation of near-eye display devices in various work-place scenarios in the next few years. The findings of our study justify further investigations in this field.

## Supporting Information

S1 File Supporting information regarding our main outcome measures. (XLSX)
